# Substrate Selectivity of the Acid-activated Glutamate/γ-Aminobutyric acid (GABA) Antiporter GadC from Escherichia coli*[Fn FN2]

**DOI:** 10.1074/jbc.M113.474502

**Published:** 2013-04-15

**Authors:** Dan Ma, Peilong Lu, Yigong Shi

**Affiliations:** From the Ministry of Education Protein Science Laboratory and the Joint Center for Life Sciences, Center for Structural Biology, Schools of Life Sciences and Medicine, Tsinghua University, Beijing 100084, China

**Keywords:** Amino Acid Transport, Glutamate, Glutamine, Membrane Proteins, Membrane Transporter Reconstitution, Acid Resistance, GadC, Membrane Antiporter, Protonation/Deprotonation, Substrate Selectivity

## Abstract

GadC, a central component of the *Escherichia coli* acid resistance system, is a Glu/GABA antiporter. A previous structural study and biochemical characterization showed that GadC exhibits a stringent pH dependence for substrate transport, with no detectable activity at pH values above 6.5. However, the substrate selectivity and the mechanism of pH-dependent transport activity of GadC remain enigmatic. In this study, we demonstrate that GadC selectively transports Glu with no net charge and GABA with a positive charge. A C-plug-truncated variant of GadC (residues 1–470) transported Gln (a mimic of Glu with no net charge), but not Glu, even at pH 8.0. The pH-dependent transport of Gln by this GadC variant was shifted ∼1 unit toward a higher pH compared with Glu transport. Taken together, the results identify the substrate selectivity for GadC and show that the protonation states of substrates are crucial for transport.

## Teaser

**Background:** GadC is a glutamate/GABA antiporter in the *Escherichia coli* acid resistance system.

**Results:** GadC selectively transports glutamate with no net charge and GABA with a positive charge. C-terminally truncated GadC transports Gln, but not Glu, at pH 7.0 or higher.

**Conclusion:** This study identifies the substrate selectivity for GadC.

**Significance:** The conclusion may be applicable to other members of the APC superfamily.

## Introduction

Virulent enteric bacteria, including *Escherichia coli* strain O157:H7, *Shigella*, *Salmonella*, and *Yersinia* (1), pose a major health threat. To survive an extremely acidic environment such as the stomach (pH 2–3), these bacteria have acquired several acid resistance systems (ARs)^3^ (2, 3) to prevent intracellular pH decrease and to maintain a relatively stable physiological pH range. Of the known ARs, the functional mechanism of AR1 remains elusive (4–7). In contrast, the mechanisms by which AR2 and AR3 function have been convincingly deciphered, each involving an amino acid antiporter/decarboxylase conjugated system. In AR2, free protons become bonded to carbon atoms through decarboxylation of Glu by GadA/GadB (8, 9) in the cytoplasm, and the reaction product GABA is exchanged with extracellular Glu through the Glu/GABA antiporter GadC (7, 10). GadC is a representative member of the APC (amino acid/polyamine/organocation) superfamily of membrane transporters (11, 12). AR3 is also composed of two key components, the Arg/agmatine antiporter AdiC (13–16) and the Arg decarboxylase AdiA (17). Similar to AR2, AR3 expels protons out of the cell through Arg decarboxylation and Arg/agmatine antiport.

We have recently reported the crystal structure of GadC, which exists in an inward facing conformation with its C-terminal domain (C-plug) blocking the transport path (18). A biochemical study showed that, compared with AdiC, GadC exhibits a much greater stringent pH dependence for substrate transport (18). WT GadC exhibits undetectable substrate transport activity at pH 6.5 and above. Intriguingly, the C-plug-truncated variant of GadC (GadC-ΔC) has considerable transport activity at pH 6.5 (18). Deletion of the C-plug in GadC shifted its pH-dependent substrate transport toward a higher pH by 0.5 pH unit, suggesting that the C-plug of GadC may regulate the pH-dependent substrate transport. In addition, GadC can efficiently transport Gln into *E. coli* cells to provide acid resistance through enzymatic release of ammonium (19). The *V*_max_ and *K_m_* values for Gln are similar to those for Glu.

**FIGURE 1.**
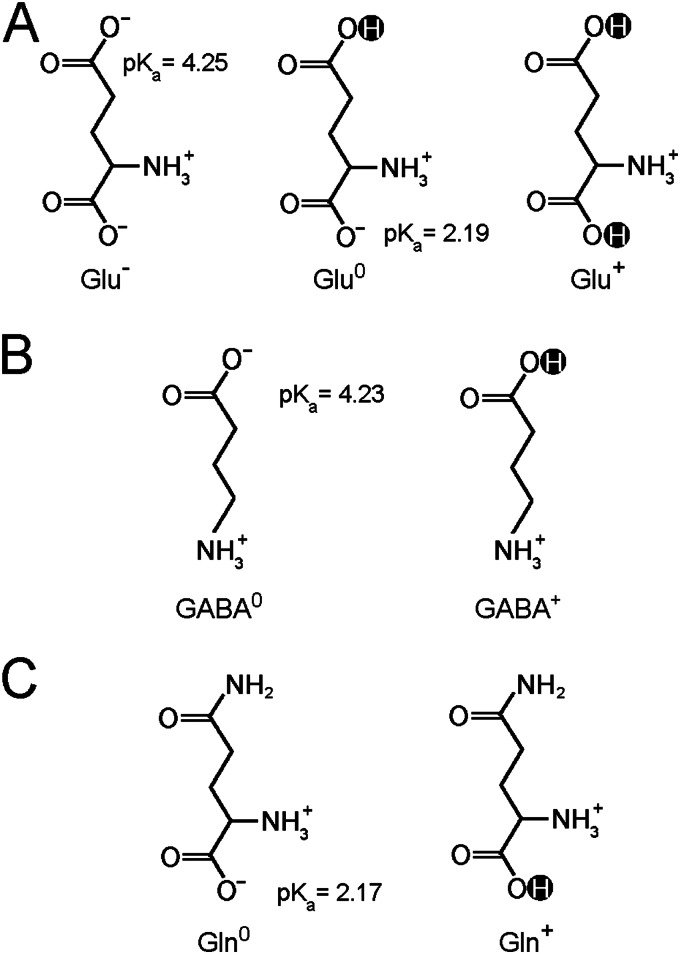
**Glu, Gln, and GABA display more than one charge state at pH 5.5.** *A*, Glu may carry one negative charge (Glu^−^), no net charge (Glu^0^), or one positive charge (Glu^+^). *B*, GABA exists in two states: with no net charge (GABA^0^) and with one positive charge (GABA^+^). *C*, Gln exists in two potential charged states: with no net charge (Gln^0^) and with one positive charge (Gln^+^).

Despite these mechanistic investigations, several crucial questions remain to be addressed. In particular, given the fact that Glu can exist in three distinct charge states (Fig. 1*A*), it is unclear whether GadC has any selectivity and, if so, which charge state(s). Other known substrates, Gln and GABA, also have more than one possible charge states (Fig. 1, *B* and *C*). Elucidation of substrate selectivity is at the center of the pH-dependent transport mechanism for GadC.

GadC functions at acidic pH and becomes inactive at neutral pH and above (18). Apparently, different pH values affect distribution of the various charge states for each substrate. With p*K_a_* values of 9.1–10.4, >99.9% of the α-amino groups of Glu/Gln and the γ-amino groups of GABA are protonated at pH 5.5. Thus, Glu may carry one negative charge (Glu^−^), no net charge (Glu^0^), or one positive charge (Glu^+^) (Fig. 1*A*). Similarly, GABA exists in two states: with no net charge (GABA^0^) and with one positive charge (GABA^+^) (Fig. 1*B*). Gln also exists in two potential charged states: with no net charge (Gln^0^) and with one positive charge (Gln^+^) (Fig. 1*C*). Among these various states, Gln^+^ and Glu^+^ require protonation of their α-carboxyl groups (p*K_a_* ∼ 2.17–2.19) and thus are unlikely to populate within living cells, where the pH value cannot drop below 3.5 (20).

Previous studies show that the exchange of Glu and GABA is strongly influenced by membrane potential in the proteoliposome-based assay; GABA efflux and Glu influx are markedly increased at a positive potential inside the proteoliposomes and decreased at negative potentials inside the proteoliposomes (18). These observations indicate that GABA carries additional positive charge(s) compared with Glu (18). Thus, there are three theoretical possibilities for Glu/GABA exchange: Glu^−^/GABA^0^, Glu^−^/GABA^+^, and Glu^0^/GABA^+^. Because Gln can be transported by GadC with high efficiency and structurally mimics the γ-carboxyl group protonated form of Glu (Glu^0^ or Glu*^+^*), we hypothesized that Glu^0^ is the primary substrate for GadC.

In this study, we demonstrate that GadC transports Glu^0^, Gln^0^, and GABA^+^ in a proteoliposome-based counterflow assay. These results indicate that the protonation states of a given substrate are crucial for transport and that side chain deprotonation of Glu and GABA at neutral pH impedes Glu/GABA exchange.

## EXPERIMENTAL PROCEDURES

### 

#### 

##### Protein Preparation

The experimental procedures were similar to those reported previously (18). A hexahistidine tag was proteolytically removed on a Ni^2+^-nitrilotriacetic acid affinity column (Qiagen) after washing with buffer containing 25 mm Tris-HCl (pH 8.0), 150 mm NaCl, 20 mm imidazole, and 0.02% *n*-dodecyl-β-d-maltopyranoside (Anatrace). GadC was eluted and further purified by gel filtration (Superdex 200 10/30, GE Healthcare). The peak fractions were collected. GadC-ΔC was prepared in the same way as the WT protein.

##### Preparation of Liposomes and Proteoliposomes

Liposomes and proteoliposomes were prepared using *E. coli* polar lipid (Avanti) as described previously (18). Liposomes and proteoliposomes were loaded with 5 mm substrate (Glu, GABA, or Gln), 150 mm KCl, and 25 mm various buffer systems: MES buffer or phosphate buffer was used at pH 5.5, MES buffer was used at pH 6.0 and 6.5, HEPES buffer was used at pH 7.0 and 7.5, and Tris buffer was used at pH 8.0 and 8.5.

##### In Vitro Transport Assay

All transport assays were carried out as described previously (18). Reactions were initiated by the addition of proteoliposomes (2 μl) to 100 μl of external buffer containing various buffer systems (25 mm), 150 mm KCl, and 50 μm unlabeled Glu plus 0.2 μm [^3^H]Glu (PerkinElmer Life Sciences) or 50 μm unlabeled Gln plus 0.2 μm [^3^H]Gln (PerkinElmer Life Sciences). The uptake of ^3^H-labeled substrate was stopped at the indicated time points. All experiments were repeated at least three times.

##### Buildup of Membrane Potential

Liposomes were loaded with 5 mm GABA or Glu and either 120 mm sodium phosphate (pH 5.5) or potassium phosphate (pH 5.5). Reactions were initiated by the addition of proteoliposomes (2 μl) to 100 μl of external buffer containing 120 mm potassium phosphate (pH 5.5) or sodium phosphate (pH 5.5), 50 μm unlabeled Gln, and 0.2 μm [^3^H]Gln, with or without 1 μg/ml valinomycin.

##### Determination of V_max_ and K_m_ Values

For determination of the *V*_max_ and *K_m_* values, the same substrates were used on both sides of proteoliposomes (Glu/Glu or Gln/Gln). The chosen time points were within the linear range of substrate accumulation: for Glu, a 1-min time point was chosen at pH 5.5, and 10-min time point was chosen at pH 6.0 and 6.5; and for Gln, 1-min time point was chosen at pH 5.5 and 6.0, and 10-min time point was chosen at pH 6.5 and 7.0. Proteoliposome preparation and the substrate transport procedure were described above.

## RESULTS

### 

#### 

##### GadC Transports Glu^0^

**FIGURE 2.**
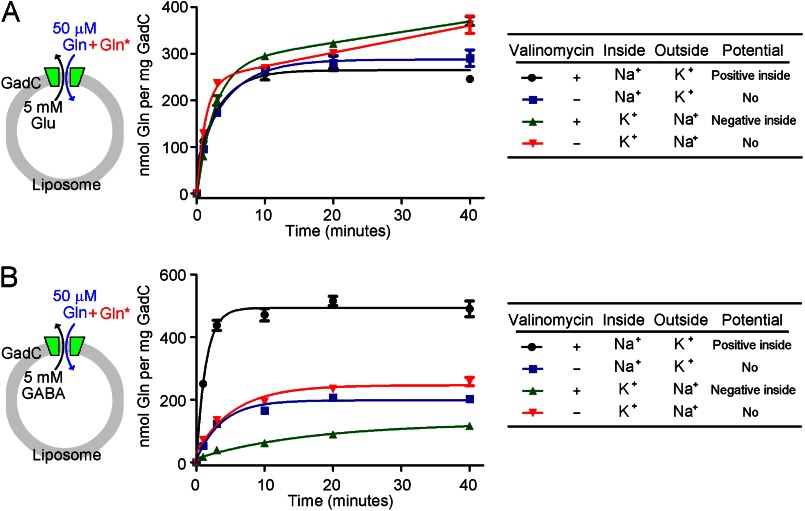
**Influence of membrane potential on substrate transport by GadC.** *A*, influence of membrane potential on the exchange of Gln and Glu. Substrate transport by WT GadC was measured in a proteoliposome-based assay at pH 5.5 in the presence or absence of valinomycin, which allows selective passage of K^+^ (but not Na^+^) ions. In this assay, the proteoliposomes were loaded with 5 mm Glu, and the external buffer contained 50 μm unlabeled Gln and 0.2 μm [^3^H]Gln. With 120 mm Na^+^ in the proteoliposome and 120 mm K^+^ in the external buffer, valinomycin allowed influx of K^+^ into the proteoliposome, generating a positive potential inside the proteoliposome. Such a potential had little effect on the influx of Gln and the efflux of Glu (compare *black* and *blue line*s). Conversely, with 120 mm K^+^ in the proteoliposome and 120 mm Na^+^ in the external buffer, valinomycin allowed efflux of K^+^ into the external buffer, resulting in a negative potential inside the proteoliposome. Such treatment had little effect on substrate transport by GadC (compare *green* and *red lines*). These results indicate that there is no net charge difference between the transport substrates Gln and Glu. *B*, influence of membrane potential on exchange of Gln and GABA. The experimental design used here was similar to that described for *A*, except that the proteoliposomes were loaded with 5 mm GABA. The positive potential inside the proteoliposome resulted in markedly enhanced substrate transport by GadC, *i.e.* increased influx of Gln and efflux of GABA (compare *black* and *blue line*s). Conversely, the negative potential inside the proteoliposome led to significantly decreased substrate transport by GadC (compare *green* and *red lines*). These results indicate that, compared with the charge state of Gln, GABA should be at least +1.

If our hypothesis that GadC transports Glu^0^ is true, then electrostatic potential across the membranes of the proteoliposome should have little effect on GadC-mediated exchange of Gln and Glu. To examine this scenario, we purified recombinant WT GadC to homogeneity and investigated the influence of membrane potential on the exchange of Gln and Glu at pH 5.5 (Fig. 2*A*). In this assay, the proteoliposomes were loaded with 5 mm Glu, and the external buffer contained 50 μm Gln and [^3^H]Gln. The inclusion of 120 mm Na^+^ within the proteoliposomes and 120 mm K^+^ in the external buffer allowed influx of K^+^ ions into the proteoliposomes in the presence of valinomycin, generating a positive inside potential. This potential had little effect on substrate transport by GadC, with temporal accumulation of Gln in the presence of the positive inside potential (Fig. 2*A*, *black line*) comparable to that in its absence (*blue line*).

Similarly, inclusion of 120 mm K^+^ inside the proteoliposomes and 120 mm Na^+^ in the external buffer allowed efflux of K^+^ ions into the external buffer in the presence of valinomycin, generating a negative inside potential. This potential had little effect on substrate transport by GadC (Fig. 2*A*, compare *green* and *red lines*). The membrane potential-independent exchange of Gln and Glu strongly suggests that both substrate molecules may carry the same amount of net charge. Gln can exist as either Gln^0^ or Gln^+^ over the pH range 0–7, whereas our prior analysis showed that GadC can transport only Glu^−^ or Glu^0^. Together, these results unambiguously demonstrate that Glu^0^, but not Glu^−^, is the substrate for GadC.

##### GadC Transports GABA^+^

The results from the above analysis, together with our previous data (18), indicate that only GABA^+^ can be transported by GadC. We sought to verify this prediction by examining the effect of membrane potential on GadC-mediated exchange of Gln and GABA in a proteoliposome-based assay at pH 5.5 (Fig. 2*B*). Under this condition, Gln exists mainly as Gln^0^, and GABA exists as GABA^0^ or GABA^+^. In this assay, the proteoliposomes were loaded with 5 mm GABA, and the external buffer contained 50 μm Gln and [^3^H]Gln. In complete agreement with our prediction, a positive potential inside the proteoliposomes significantly enhanced the influx of Gln and the efflux of GABA (Fig. 2*B*, compare *black* and *blue lines*). The initial exchange rate of Gln and GABA in the presence of the positive inside potential was ∼4-fold faster than that in its absence. The total accumulation of Gln at the 40-min time point in the presence of the positive inside potential (Fig. 2*B*, *black line*) was ∼2.5-fold of that in its absence (*blue line*).

Conversely, the negative potential inside the proteoliposome had a negative impact on the influx of Gln and the efflux of GABA (Fig. 2*B*, compare *green* and *red lines*). Both the initial exchange rate of Gln and GABA and the total accumulation of Gln at the 40-min time point in the presence of the negative inside potential (Fig. 2*B*, *green line*) were less than half of those in its absence (*red line*). These results reveal that GABA carries additional positive charge compared with Gln^0^ and confirm our prediction that GABA^+^ is the transport substrate for GadC.

##### pH-dependent Transport of Glu and Gln by GadC-ΔC

WT GadC exhibits undetectable transport activity at pH 6.5 and above (18). Removal of the C-plug in GadC (GadC-ΔC) allowed restoration of transport at pH 6.5 and shifted the pH-dependent substrate transport by 0.5 pH unit toward a higher pH. However, at pH 7.0 and above, the transport activity of GadC-ΔC for Glu is completely abolished (18). Given the observation that GadC transports only Glu^0^, we hypothesized that the abolished transport activity is due at least in part to the deprotonation of Glu at neutral pH. If this hypothesis is true, then transport of Gln should occur at pH 7.0 and above, under which condition Gln exists predominantly as Gln^0^.

**FIGURE 3.**
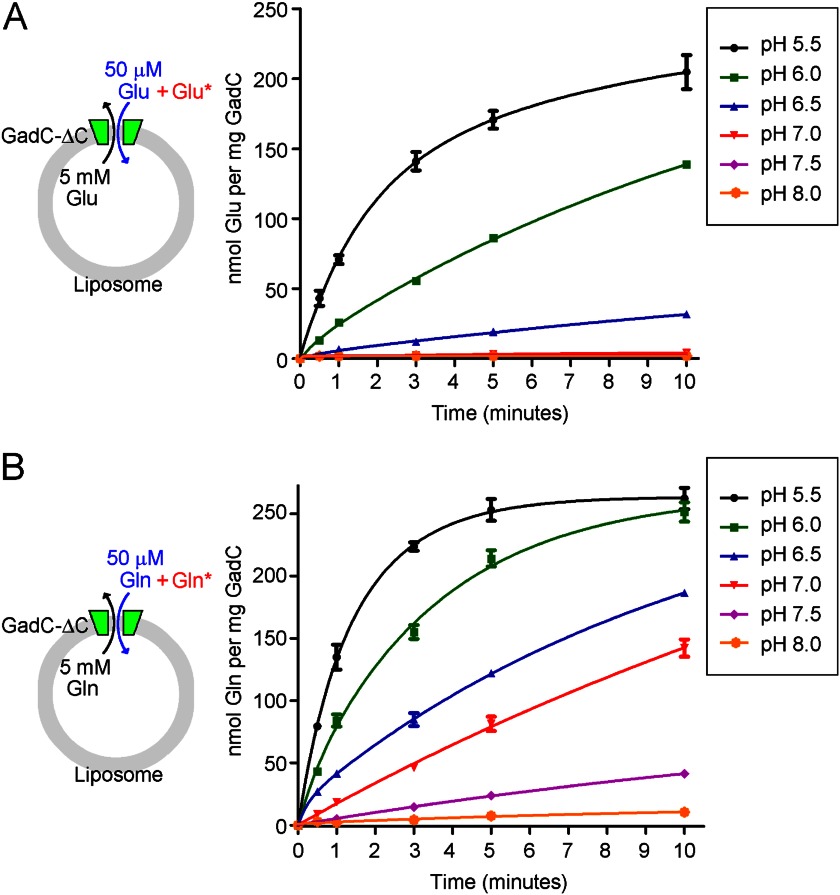
**GadC-ΔC exhibits pH-dependent transport of Glu and Gln.** *A*, the transport of Glu was robust at pH 5.5 and rapidly decreased with increasing pH values, with no detectable transport activity at pH 7.0 and above. The transport of Glu by the C-plug-deleted GadC variant (GadC-ΔC) was measured in a proteoliposome-based assay at different pH values. In this assay, the proteoliposomes were loaded with 5 mm Glu, and the external buffer contained 50 μm unlabeled Glu and 0.2 μm [^3^H]Glu. *B*, the transport of Gln was still measurable at pH 8.0. The transport of Gln by GadC-ΔC decreased with increasing pH values, but the rate of decrease was markedly slower than that for transport of Glu.

To examine this scenario, we purified GadC-ΔC to homogeneity and investigated the transport of Gln by GadC-ΔC at six different pH values (pH 5.5, 6.0, 6.5, 7.0, 7.5, and 8.0) using the proteoliposome-based assay. pH-dependent transport of Glu was performed as a control experiment (Fig. 3*A*). The accumulation of Glu within 10 min at pH 5.5 was robust, and the level of Glu transport sharply decreased with increasing pH values. At pH 7.0 and above, there was no detectable accumulation of Glu (Fig. 3*A*). In sharp contrast, Gln transport by GadC-ΔC was quite efficient at pH 7.0 (Fig. 3*B*, *red line*) and still detectable at pH 7.5 and 8.0 (Fig. 3*B*, *purple* and *orange lines*). Transport of Gln by GadC-ΔC also decreased with increasing pH values, but the rate of decrease was markedly slower than that for transport of Glu. The accumulation of Gln at the 10-min time point at pH 6.0, 6.5, 7.0, 7.5, and 8.0 was ∼96, 71, 54, 16, and 4% of that at pH 5.5 (Fig. 3*B*).

**FIGURE 4.**
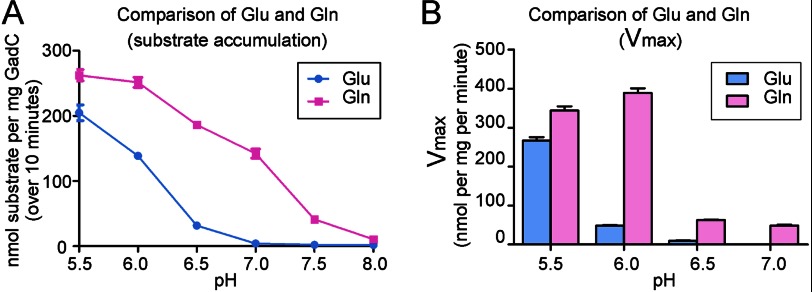
**pH-dependent transport of Gln by GadC-ΔC is shifted ∼1 pH unit toward a higher pH compared with that of Glu.** *A*, comparison of the substrate accumulation of Glu and Gln. For Glu, GadC-ΔC exhibited no detectable transport activity at pH above 7.0. In contrast, GadC-ΔC could transport Gln even at pH 8.0. *B*, comparison of *V*_max_ values of GadC-ΔC for Glu and Gln at different pH values.

We compared the accumulation of Glu and Gln over 10 min at different pH values (Fig. 4*A*). The data show that pH-dependent accumulation of Gln by GadC-ΔC was shifted ∼1 unit toward a higher pH compared with that of Glu (Fig. 4*A*). To further characterize the pH-dependent substrate transport, we compared the activities of GadC-ΔC for Glu and Gln transport under identical sets of experimental conditions. All transport rate *versus* substrate concentration curves were fitted using the Michaelis-Menten equation, and the resulting maximal transport activities (*V*_max_) for Glu and Gln at different pH values were determined (Fig. 4*B* and supplemental Fig. 1). The *V*_max_ values for Glu at pH 6.0 and 6.5 were ∼21 and 4% of that at pH 5.5, and the transport activity for Glu at pH 7.0 was undetectable. In contrast, the *V*_max_ values for Gln at pH 6.0, 6.5, and 7.0 were ∼114, 18, and 14% of that at pH 5.5, respectively. These results are consistent with the substrate accumulation results shown in Fig. 4*A*. Thus, the pH-dependent transport of Gln by GadC-ΔC is shifted toward a higher pH by 1 unit compared with that of Glu.

## DISCUSSION

**FIGURE 5.**
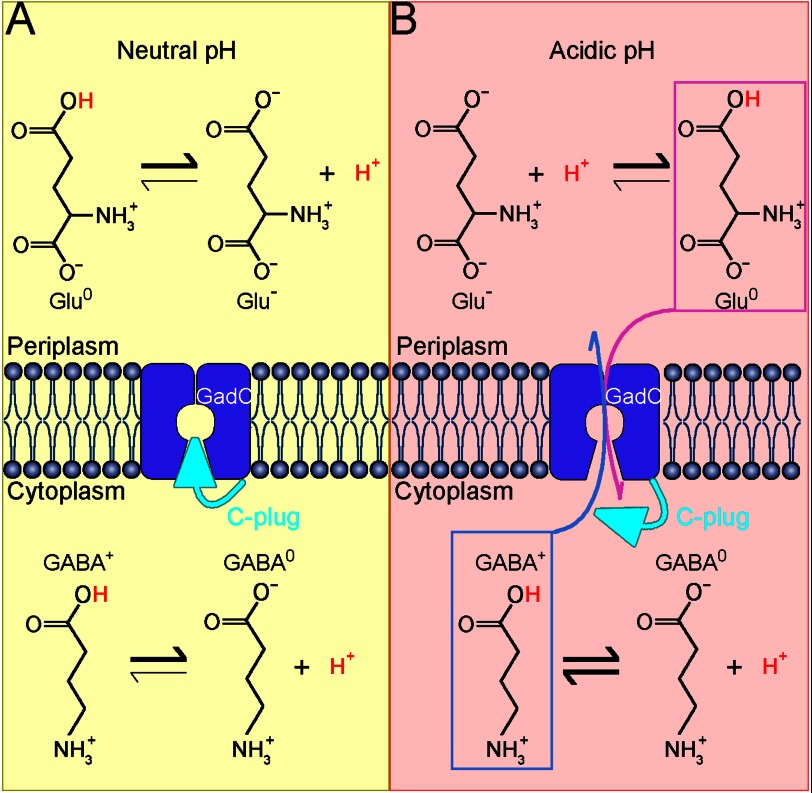
**Proposed mechanism of pH-dependent substrate transport by GadC.** *A*, GadC is inactive at neutral pH. Most of the substrate molecules (Glu and GABA) are deprotonated, thus are unfavorable for transport. In addition, the substrate transport path is blocked by the C-plug of GadC. Under these conditions, no substrate exchange occurs. *B*, GadC is activated at acidic pH. When the extracellular pH is extremely acidic (pH 2∼3), the cytoplasmic pH in *E. coli* drops to a value between 3.5 and 5.0. In the cytoplasm, GABA exists in two forms, with no net charge (GABA^0^) and with one positive charge (GABA^+^), whereas the majority of Glu in the extracellular space carries no net charge (Glu^0^). At acidic pH, the C-plug of GadC may be displaced, allowing influx of Glu^0^ and efflux of GABA^+^.

Our results demonstrate that GadC exchanges Glu^0^ with GABA^+^, or Gln^0^ with Glu^0^. Our experimental findings also indicate that side chain deprotonated states of Glu and GABA (Glu^−^ and GABA^0^) are selected against by GadC. On the basis of these results, we propose a working model to illustrate the mechanism of the pH-dependent transport by GadC in *E. coli* (Fig. 5). In a neutral pH environment that is favorable to survival (Fig. 5*A*), the cytoplasm of *E. coli* is likely maintained at neutral pH. Most of the substrates exist in their deprotonated states (Glu^−^ and GABA^0^), which are not substrates for GadC. In addition, the C-plug of GadC blocks the transport path. Under these conditions, no substrates exchange occurs. When *E. coli* is exposed to an extremely acidic environment (such as pH 2), the cytoplasm also becomes considerably acidic (such as pH 4.2 (20)) (Fig. 5*B*). At this pH, the C-plug of GadC should be dislodged, and the transport path is open. In the stomach (pH ∼2), ∼99% of the Glu side chain (p*K_a_* ∼ 4.25) is protonated, and roughly half of the extracellular Glu exists as Glu^0^. Glu^0^ will be transported into the cell by GadC. The imported Glu is decarboxylated to generate GABA (p*K_a_* ∼ 4.23, α-carboxyl group). Almost equal amounts of GABA^0^ and GABA^+^ are present in the cytoplasm, but only GABA^+^ can be exported by GadC.

The pH-dependent transport of GadC ensures that substrate exchange can be efficiently activated only in an acidic environment, preventing an unnecessary waste of energy. Intriguingly, Gln transport by GadC-ΔC at pH 8.0 was much less active than at pH 5.5 (Fig. 4). This result indicates that pH-dependent substrate protonation does not fully explain the pH-dependent substrate transport by GadC-ΔC and further suggests the presence of a pH sensor in the GadC antiporter itself. This putative pH sensor likely forces selective closure of the transport path of GadC at neutral and basic pH. The exact nature of this pH-sensing mechanism awaits further biochemical and structural investigations.

It should be noted that, although our *in vitro* transport assays were performed at pH 5.5, the conclusions should be applicable to the physiological conditions. Our conclusions may also have significant implications for GadC homologs such as AdiC and CadB and possibly other members of the APC superfamily.

## Supplementary Material

Supplemental Data

## Footnotes

3 The abbreviation used is:

AR: acid resistance system.

## Addendum

While this manuscript was in the final stage of preparation, Tsai et al. (21) reported a similar study. We note that the conclusions in that report are generally consistent with ours, although the detailed experimental approaches are different. Tsai et al. identified Glu^−^ and Glu^0^ as potential substrates for GadC, whereas our analysis suggests that only Glu^0^ may serve as the substrate.
