# Supplementary material for: Substrate Selectivity of the Acid-activated Glutamate/γ-Aminobutyric acid (GABA) Antiporter GadC from Escherichia coli
Source: J Biol Chem. 2013 Apr 15;288(21):15148–53. doi: 10.1074/jbc.M113.474502 (PMC3663535; doi:10.1074/jbc.M113.474502)
Supplement: Supplemental Data [file supp_288_21_15148__index.html]

Substrate Selectivity of the Acid-activated Glutamate-GABA Antiporter GadC from E. coli — Substrate Selectivity of the Acid-activated Glutamate/γ-Aminobutyric acid (GABA) Antiporter GadC from Escherichia coli — Substrate Selectivity of GadC — Supplemental Data 

# Substrate Selectivity of the Acid-activated Glutamate/γ-Aminobutyric acid (GABA) Antiporter GadC from *Escherichia coli*

## Supplemental Data

**Files in this Data Supplement:**

- Supplementary Figure 1 (.pdf, 106 KB) - The transport activities of GadC-&#x26;Delta;C for Glu and Gln.
