# Supplementary material for: Substrate Selectivity of the Acid-activated Glutamate/γ-Aminobutyric acid (GABA) Antiporter GadC from Escherichia coli
Source: J Biol Chem. 2013 Apr 15;288(21):15148–53. doi: 10.1074/jbc.M113.474502 (PMC3663535; doi:10.1074/jbc.M113.474502)
Supplement: Supplemental Data [file supp_M113.474502_jbc.M113.474502-1.pdf]

Supplementary Figure Legend

Supplementary Fig. 1 The transport activities of GadC-ΔC for Glu and Gln. All transport rate versus substrate concentration curves were fitted using Michaelis-Menten equation, and the resulting maximal transport activity (V_{max}) for Glu (panel A) and Gln (panel B) at different pH values were determined.

Supplementary Fig. 1

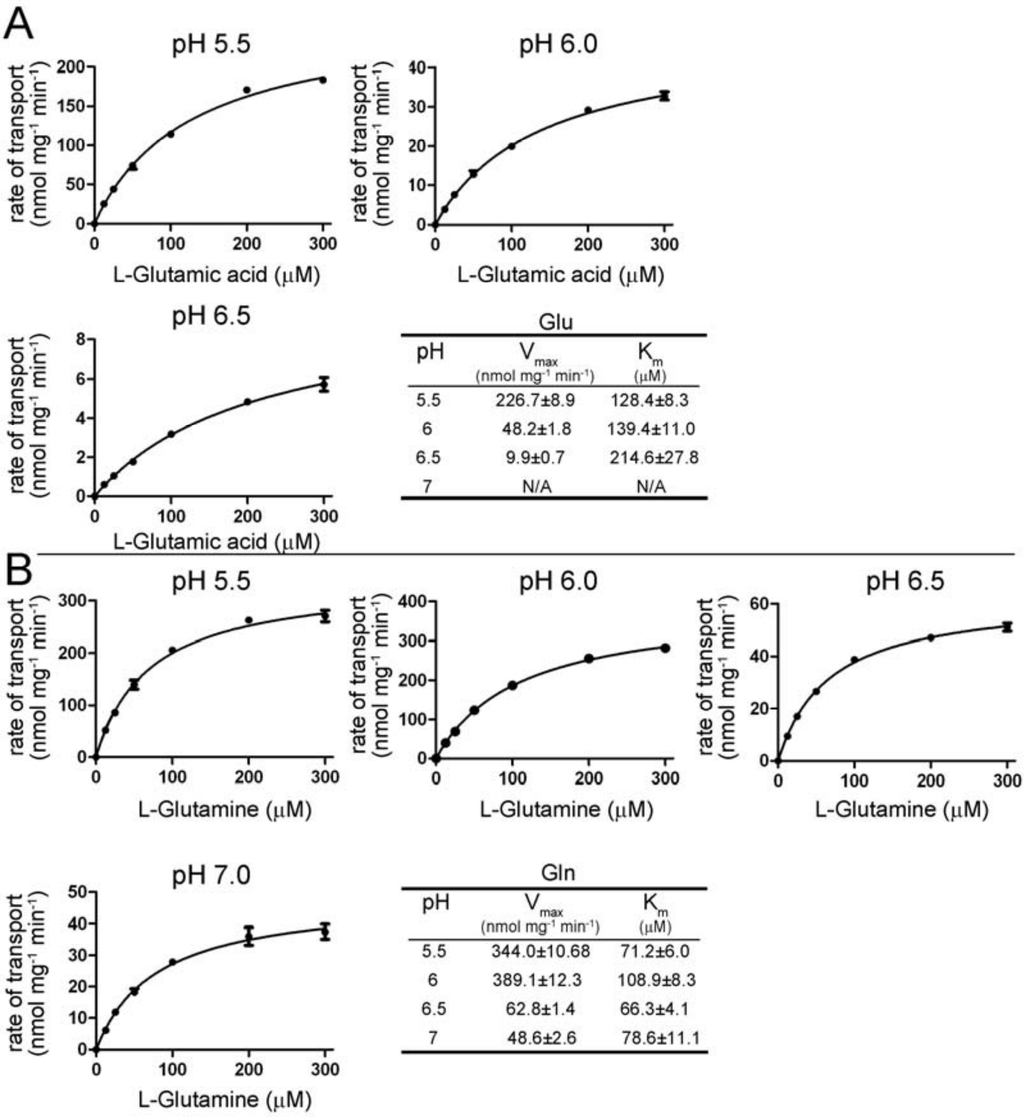

Glu

pH	V_{max} (nmol mg^{-1} min^{-1})	K_m (μM)
5.5	226.7±8.9	128.4±8.3
6	48.2±1.8	139.4±11.0
6.5	9.9±0.7	214.6±27.8
7	N/A	N/A

Gln

pH	V_{max} (nmol mg^{-1} min^{-1})	K_m (μM)
5.5	344.0±10.68	71.2±6.0
6	389.1±12.3	108.9±8.3
6.5	62.8±1.4	66.3±4.1
7	48.6±2.6	78.6±11.1
